# Finger prosthesis: A novel way to restore the form, function, and esthetics

**DOI:** 10.1002/ccr3.8147

**Published:** 2023-11-02

**Authors:** Nagaveni S. Somayaji, Pallawi Sinha, Jitendra Sharan, Jagadish Prasad Rajaguru, Anand Marya

**Affiliations:** ^1^ Department of Prosthodontics Hi‐Tech Dental College and Hospital Bhubaneswar Odisha India; ^2^ Department of Dentistry All India Institute of Medical Sciences Bhubaneswar India; ^3^ Department of Oral and Maxillofacial Pathology Hi‐Tech Dental College and Hospital Bhubaneswar Odisha India; ^4^ Faculty of Dentistry, Department of Orthodontics and Dentofacial Orthopedics University of Puthisastra Phnom Penh Cambodia

**Keywords:** digit prosthesis, finger prosthesis, mechanical retention, RTV silicones, traumatic amputation

## Abstract

**Key Clinical Message:**

A well‐customized prosthesis with a life‐like esthetic and function are the primary determining factors for its acceptance and success. RTV silicones can prove to be very effective and useful in fabricating such prosthesis.

**Abstract:**

Complete or partial finger amputations impact an individual's psychological and physical well‐being and are considered the most frequently observed pattern of hand loss. A customized prosthesis offers the patient rehabilitative, functional, and psychological advantages. Retention is the key to the success of such prosthetic restoration. The present case report describes a novel technique that utilizes passive vacuum fit and mechanical retention to restore controlled function movements as a metal wire framework. The procedure was economical, most importantly, produced life‐like anatomy of the missing digit and restored the function to some extent.

## Graphical abstract

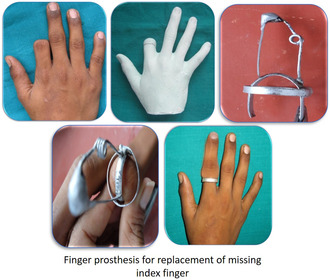

## 1 INTRODUCTION

Any deformity or loss of the body part may cause psychological and emotional disturbances to the patient.^1^, ^2^ Loss of finger/s can be secondary to congenital disorders, trauma, or excision of neoplastic conditions. Digit avulsion injuries secondary to personal accidents remain the single most etiology for the loss of digit/s.^3^, ^4^ Traumatic amputation of the finger/s depicts a serious insult that greatly impairs the hand's function and affects the patient's quality of life (QoL), making social integration difficult. Further, the active functions of the hand, such as prehensile activities in grip, grasp, transferring, and absorbing of the forces, get compromised depending on the extent and severity of the loss of digit/s.^5^ Thus, the patient's expectations of returning to normalcy are often compromised. Surgical techniques, such as microvascular surgery, to restore the avulsed digit if the patient is brought to the consultant within the shortest possible time after the incidence will increase the survival rate of the avulsed digit/s.^6^, ^7^ But, in certain cases, reconstruction is either unsuccessful or not advisable, and in such a scenario patient is provided with a prosthesis that offers a great psychological and physical boost.

Restoring the missing part of the hand by prosthesis often offers psychological, functional, and rehabilitative advantages. Custom‐made life‐like prostheses tend to eliminate the trauma secondary to the handicapping condition, thus, offer true psychological therapy. Thus, this case report aims to present a novel technique for fabricating a silicon finger prosthesis by incorporating a metal framework, which was critically designed to provide passive function and retention.

## 2 CASE HISTORY

**FIGURE 1:**
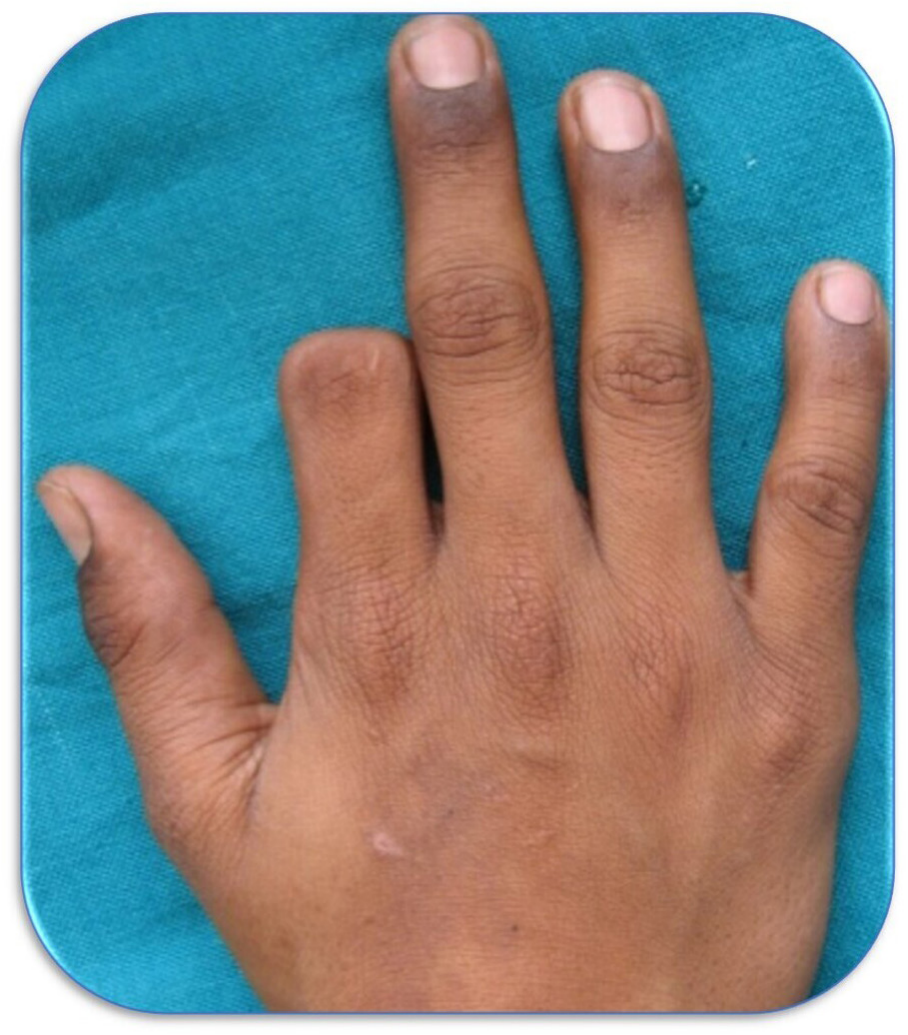
Pre‐rehabilitation view of the dorsal aspect of the right hand.

A 17‐year‐old boy with his father reported to the Department of Prosthodontics & Crown and Bridge for rehabilitation of his right partially missing index finger that he had lost 6 years back. According to the patient's father, the patient injured his finger with a single‐edge sickle. He was taken to the surgeon for corrective surgery, who performed microvascular surgery. However, after 4 days, the reimplanted part of the finger showed signs of necrosis; thus, the surgical site was re‐explored. Following this, the reimplanted finger part was removed and healed without complications. At the time of clinical assessment, the patient was well‐oriented and had no history of any medical disorders. The partial amputation involved the middle and distal phalanx of the right index finger (Figure 1). A detailed assessment of the skin around the amputated index finger revealed thickened skin with no signs of inflammation or infection. When questions were asked regarding his perception of the missing finger, he was trying to hide his right hand and said that loss of finger makes him feel socially and functionally deprived. The patient and his father were very keen to get the missing finger part. Informed consent was obtained before the beginning of the treatment.

Various treatment options were given to the patient for the finger prosthesis, such as finger prosthesis with a glove‐like outline, finger ring or metal framework, or finger prosthesis retained by an implant. After considering the cost and other factors, the patient opted for a metal framework retained prosthesis, providing some passive function. Fabrication of the finger prosthesis involved the following sequential steps:

### 2.1 Impression of the amputated finger and fabrication of working cast

**FIGURE 2:**
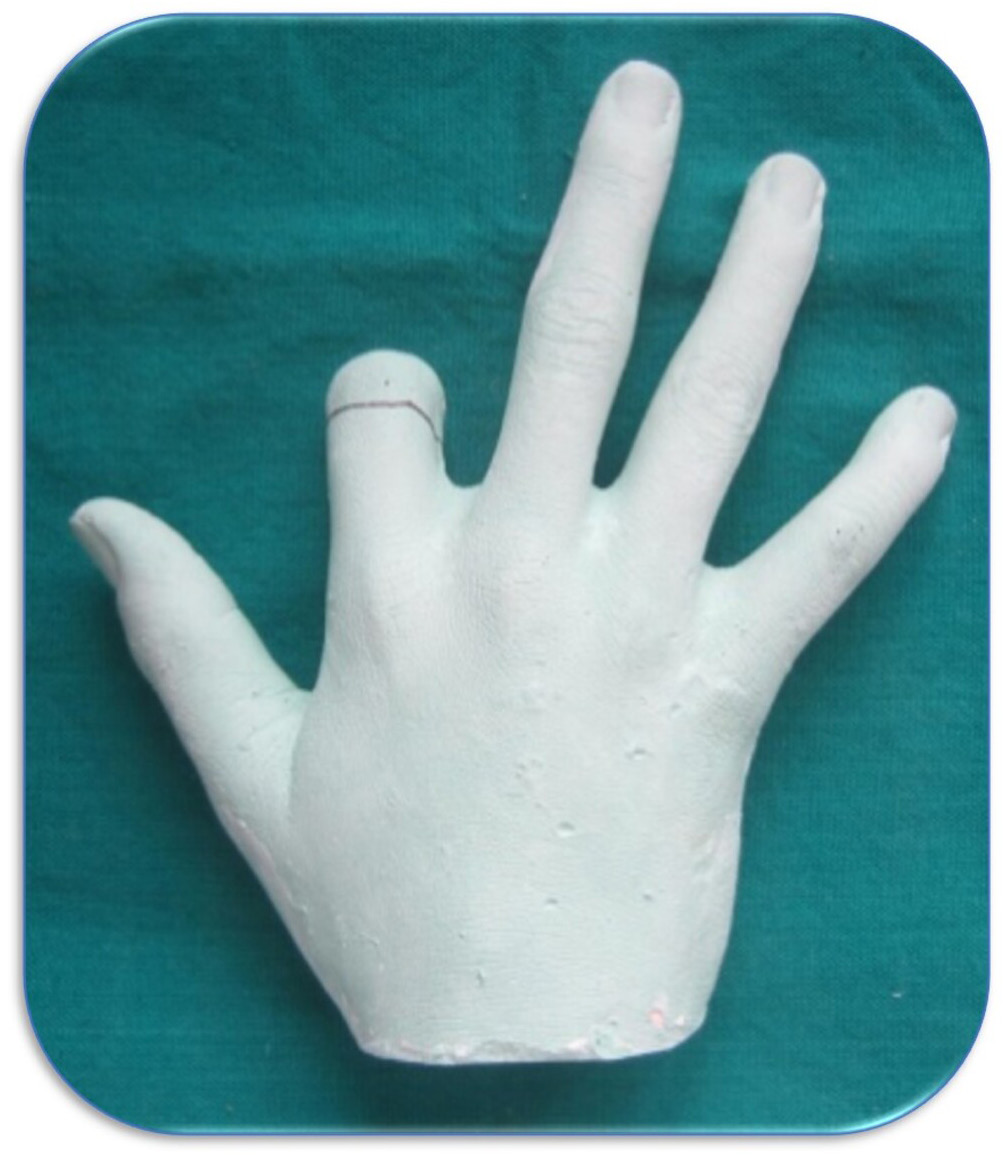
Stone model of the defect.

The impression of the hand was made in a cardboard box using alginate (Zelgan 2002, Dentsply, India). A layer of dental plaster (Kaldent, Kalabhai Karson, India) was poured over the alginate to provide the body. The alginate impression was poured with dental stone (Kalrock, Kalabhai Karson, India) to get the working cast (Figure 2).

### 2.2 Construction and working of the metal framework

**FIGURE 3:**
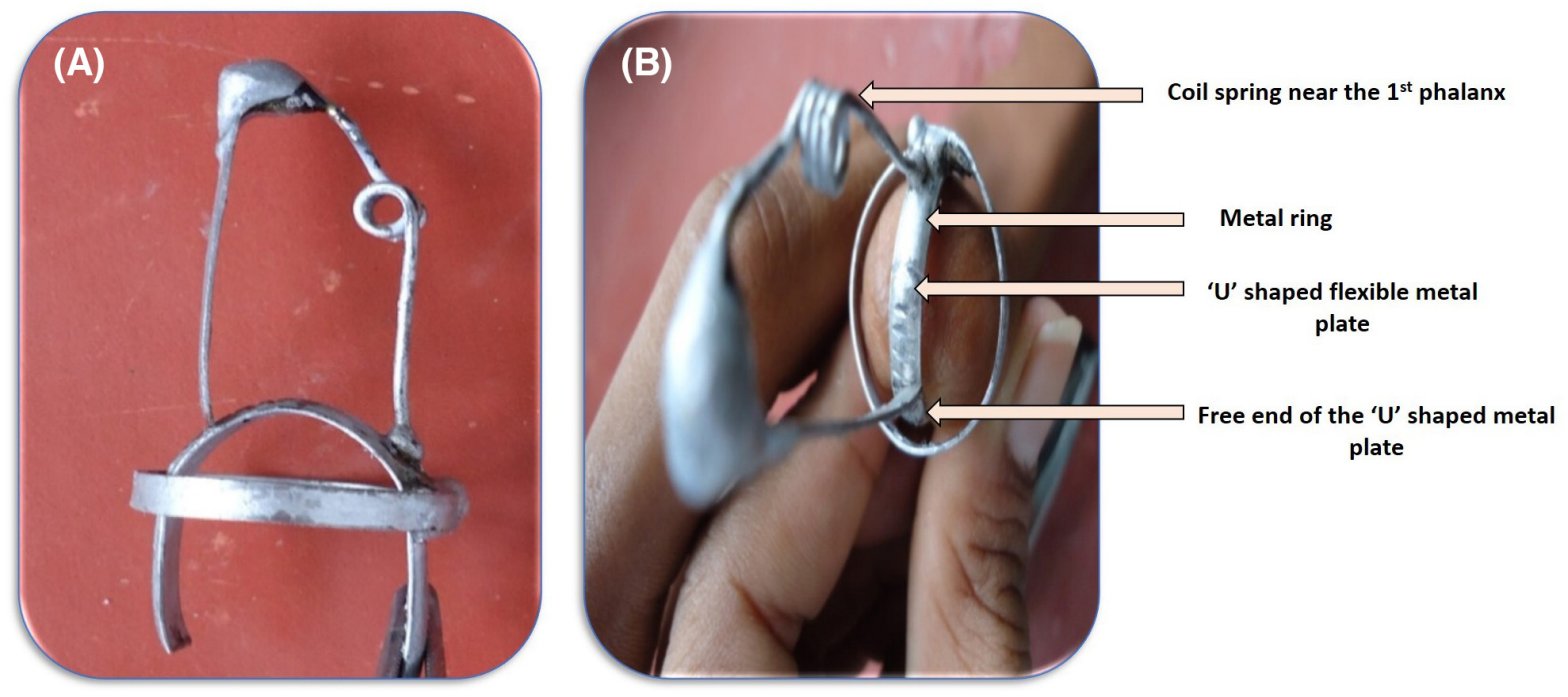
Metallic framework work of the prosthesis. (A) Outline, and (B) Components.

The metal framework consists of a metal ring that fits exactly over the amputated phalanx. It had a dual function first to provide the retention of the prosthesis and holds the different parts of the metal framework together. A rigid wire with a spring was attached to this metal ring at one end. This spring was placed at the first joint of the finger, that is, the first phalanx. Further, a U‐shaped flexible metal plate of 2 mm width with groves on the outer surface was soldered on one side while the other was kept free (Figure 3A,B). This U‐shaped metal plate lies exactly over the amputated end of the finger. When this metal framework is made to lock, it tends to bend the finger's first joint, and when it is unlocked, the finger becomes straight. The strip was unlocked by pressing the free end of the U‐shaped metal plate.

### 2.3 Wax pattern

**FIGURE 4:**
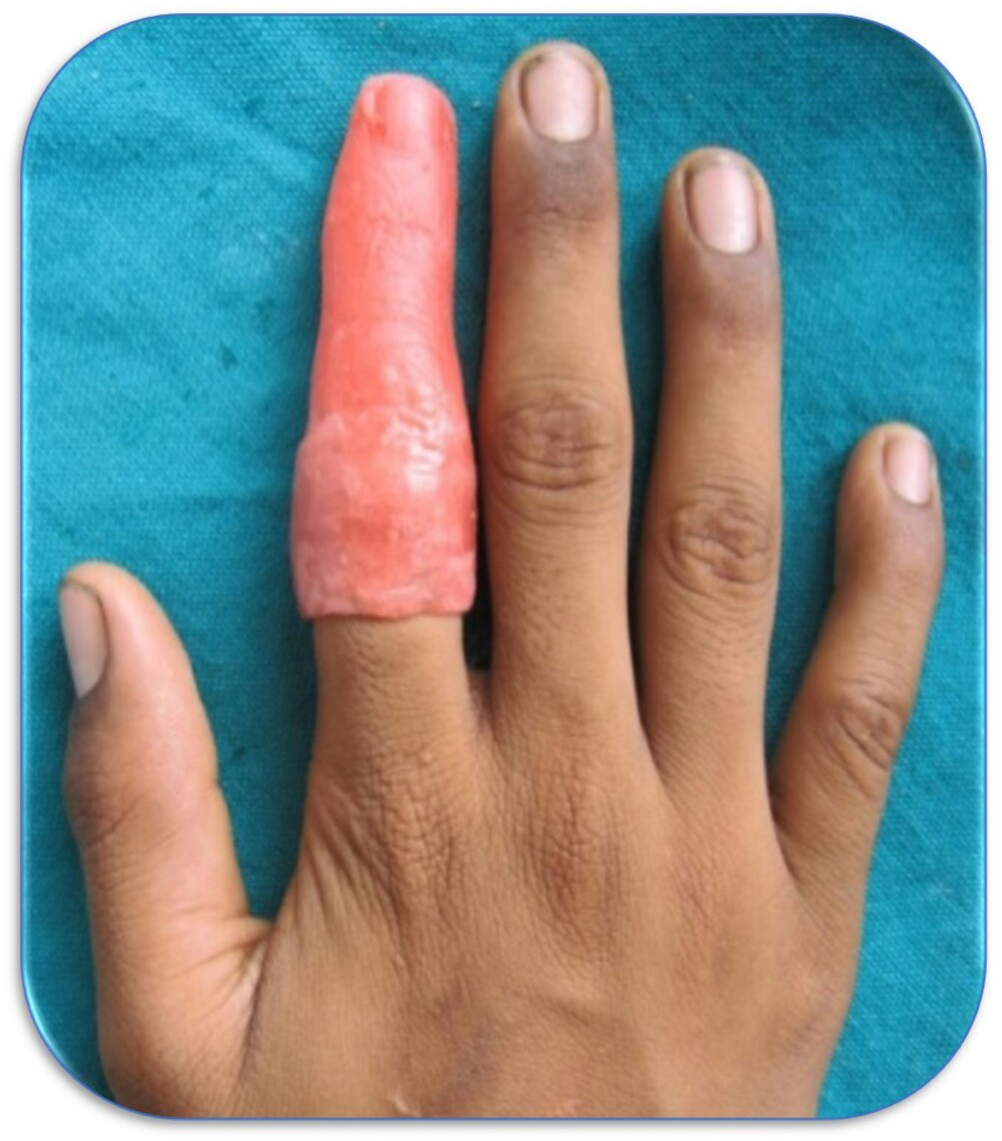
Prepared wax pattern in trial (try in).

An impression was made from the patient's counterpart normal digit using heavy body addition silicon impression material (Reprosil, Dentsply Caulk, Milford, DE, USA). Using this impression, a wax pattern is sculpted. Carving of the wax pattern was done in such a way as to incorporate the normal lateral outline of the finger in the physiologically relaxed position. Try in of the fabricated wax pattern was tried on the amputated finger and was found to be satisfactory for size, shape, retention, and minute details (Figure 4).

### 2.4 Flasking of the wax pattern

**FIGURE 5:**
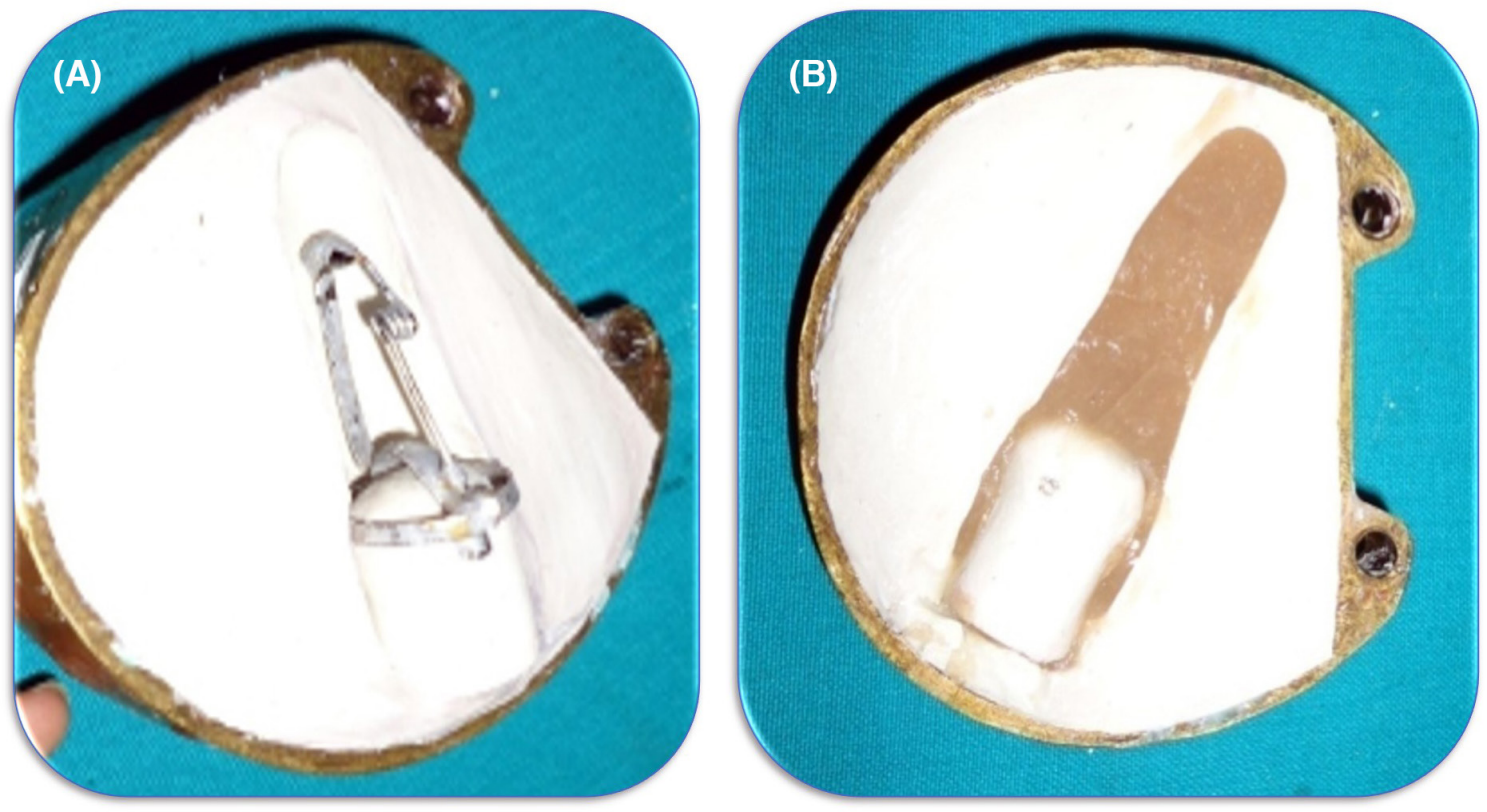
(A) Flasking of the prosthetic metal frame and (B) Post‐curing of the prosthesis.

**FIGURE 6:**
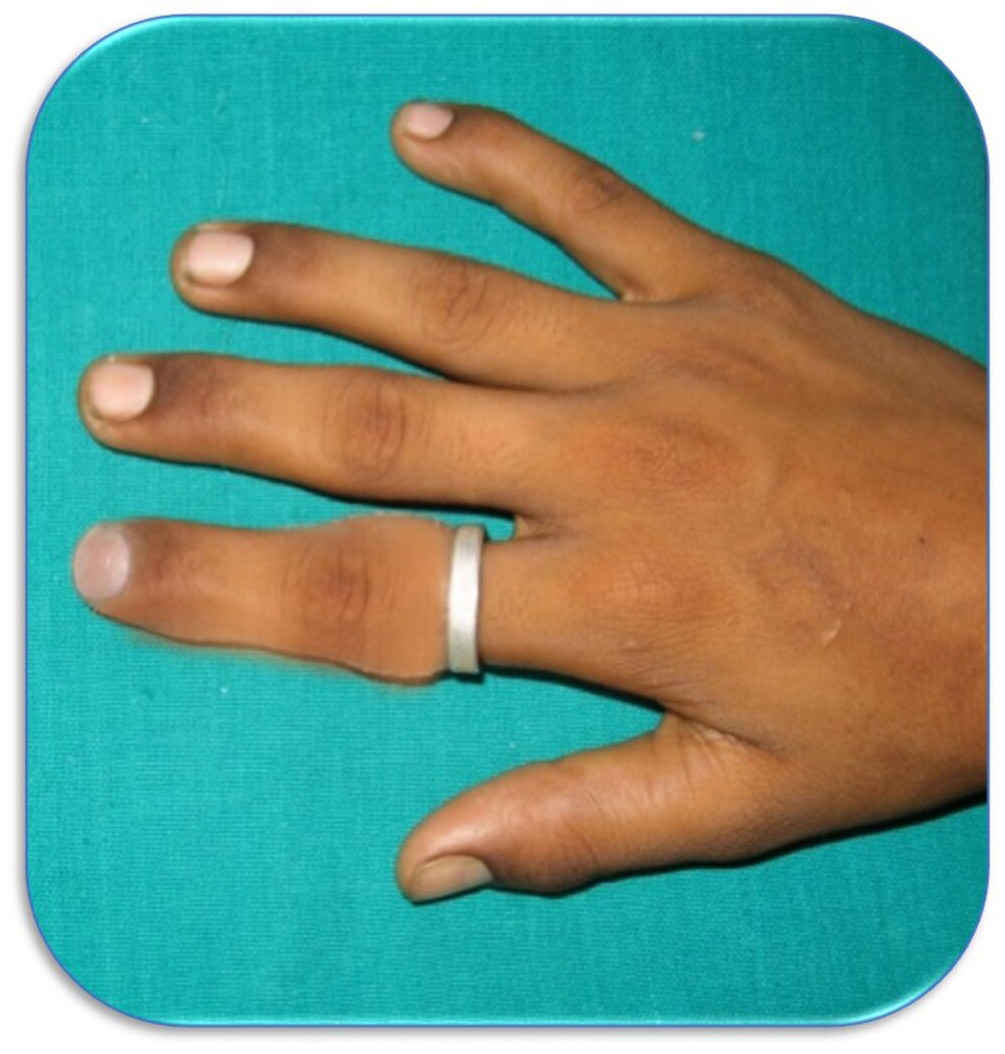
Post‐rehabilitation view of the dorsal aspect of the right hand.

The wax pattern was invested into a flask. Two pour technique was used during the flasking procedure. De‐waxing was carried out, and the metal framework was incorporated into the flask before packing the flask (Figure 5A). Packing of the room temperature vulcanizing Silicon (RTV Silicon) was done in 2 phases to match the dorsal and ventral surface of the hand (Figure 5B). The silicon material used was room temperature vulcanizing, so it was allowed to cure for 24 h. The finger prosthesis was finished, polished, and delivered to the patient (Figure 6). The patient and his father were happy and satisfied with the prosthesis.

### 2.5 Postdelivery instructions and follow up

Following the prosthesis delivery patient was informed about the instructions regarding its maintenance. Home care instructions for the prosthesis in the form of the use of an ultrasoft toothbrush along with mild soap solution and warm water irrigant were explained to him. After 1 month, he was recalled to evaluate the finger prosthesis's functioning and color stability. This has also helped us to assess the patient's satisfaction with the prosthesis, which was positive. During the recalled visit, the skin around the prosthesis remained healthy, and the prosthesis showed optimal retention and functioning.

## 3 DISCUSSION

In most cases, restoring the missing body part with the prosthesis and achieving the optimal form and function remains a great challenge to the treating clinician. In most patients, replacing the missing body part with the prosthesis eliminates psychological trauma, which is secondary to the constant reminder of the handicapping condition. Autologous reconstruction of the missing body part is preferred. Still, a customized prosthesis is preferred for various conditions, such as previously attempted but failed microvascular/plastic surgery, external beam radiotherapy, patient's wish, etc. Larcher et al. have suggested that to provide an excellent esthetic and stable point for light grasp by the finger prosthesis, 1 cm of the mobile phalanx should be available.^8^ In our case, this suggestion was considered and used for a better functional outcome. RTV silicones copy natural hand in detail; the material is supple and not subjected to ordinary thermal damage or ink stains. The metal framework incorporated in the present case provides the necessary retention for the prosthesis apart from providing some degree of passive function due to combining the spring with the metal framework. The vertical plate on the framework's inner aspect fits into the groves and provides a mechanical lock, enhancing the prosthesis's retention.

## 4 CONCLUSION

In patients with the missing finger, the custom‐made prosthesis restores the form, function, and esthetics, thus enhancing the personality. A well‐customized prosthesis with a life‐like esthetic and function are the primary determining factors for its acceptance and success. A simple but effective method using RTV silicones, a metal framework with a spring incorporated, was attempted, and found to be effective for replacing the missing finger.

## AUTHOR CONTRIBUTIONS

**Nagaveni S Somayaji:** Conceptualization; data curation; formal analysis; funding acquisition; investigation; visualization; writing – original draft; writing – review and editing. **Pallawi Sinha:** Funding acquisition; investigation; methodology; project administration; validation; visualization; writing – original draft; writing – review and editing. **Jitendra Sharan:** Data curation; formal analysis; funding acquisition; investigation; methodology; supervision; validation; visualization; writing – original draft; writing – review and editing. **Jagadish Prasad Rajaguru:** Funding acquisition; investigation; methodology; validation; visualization; writing – original draft; writing – review and editing. **anand marya:** Resources; supervision; validation; visualization; writing – original draft; writing – review and editing.

## FUNDING INFORMATION

None.

## CONFLICT OF INTEREST STATEMENT

None.

## ETHICS STATEMENT

This case report did not require review by the Ethics Committee.

## CONSENT

Written informed consent was obtained from the patient to publish this case report. **GUARANTOR** All the authors have read and approved the manuscript. I will act as guarantor and correspond with the journal on behalf of all the contributors.

## Data Availability

Any data related to the manuscript can be provided on reasonable request.
